# Effect of Reconstructive Procedures of the Extracranial Segment of the Carotid Arteries on Damage to the Blood–Brain Barrier

**DOI:** 10.3390/ijerph19106210

**Published:** 2022-05-20

**Authors:** Piotr Terlecki, Stanisław Przywara, Karol Terlecki, Dariusz Janczak, Maciej Antkiewicz, Tomasz Zubilewicz

**Affiliations:** 1 Department of Vascular Surgery and Angiology, Medical University of Lublin, 20-078 Lublin, Poland; piotrterlecki@interia.pl (P.T.); stanley.przywara@wp.pl (S.P.); tomzubil@poczta.onet.pl (T.Z.); 2 Department of Vascular Surgery, General and Transplant Surgery, Medical University in Wroclaw, 50-355 Wroclaw, Poland; dariusz.janczak@op.pl (D.J.); maciej.antkiewicz@gmail.com (M.A.)

**Keywords:** carotid artery stenosis, carotid artery endarterectomy, carotid angioplasty, blood–brain barrier, stroke, metalloproteinases, claudin-1, occludin, aquaporin-4, S100β protein

## Abstract

Introduction: Endarterectomy and angioplasty of the internal carotid artery are surgical measures for the prevention of ischemic stroke. Perioperative complications are caused by concomitant embolism and reperfusion syndrome leading to damage of the blood–brain barrier. Methods: The study included 88 patients divided into two groups, depending on the surgical technique used: internal carotid artery endarterectomy (CEA), 66 patients, and percutaneous carotid angioplasty and stenting (CAS), 22 patients. Blood was drawn 24 h before surgery, as well as 8, 24, and 48 h post-surgery. The assessment of damage to the blood–brain barrier was based on the evaluation of the concentration of claudin-1 and occludin, aquaporin-4, the measurements of the activity of metalloproteinase-2 (MMP-2) and -9 (MMP-9), and the assessment of central nervous system damage, measured by changes in the blood S100β protein concentration. Results: A significant increase in the concentration of the blood–brain barrier damage markers and increased MMP-2 and MMP-9 activity were found in patient blood. The degree of damage to the blood–brain barrier was higher in the CEA group. Conclusions: The authors’ own research has indicated that revascularization of the internal carotid artery may lead to damage to the central nervous system secondary to damage to the blood–brain barrier.

## 1. Introduction

Stroke is the most common cause of severe disability in adults and the third leading cause of death, after heart disease and cancer, in highly industrialized countries [1,2]. Approximately 20–30% of cerebral ischemic strokes are caused by advanced atherosclerotic lesions that cause internal carotid stenosis, but only 5% require interventional treatment. The degree of stenosis in the artery and the function of collateral circulation through the circle of Willis are critical [3,4].

Damage to the brain during ischemic stroke is mainly caused by hypoxia of the nerve cells [5]. Inflammatory reactions in parenchyma have also been reported, initiated by its hypoxia. Other pathological lesions include damage to the blood–brain barrier (BBB). The main factors damaging the BBB are intercellular matrix metalloproteinases released by inflammatory cells, among others, cells damaging the basal membrane, pro-inflammatory factors and free radicals secreted by inflammatory cells, as well as by mechanical factors and hypoxia damaging the vascular endothelium. Cerebrovascular edema occurs, which causes swelling of the nerve tissue and increased intracranial pressure [6,7].

The research data suggest that both intraoperative microembolism and reperfusion syndrome are conducive to central nervous system injury through BBB damage. These factors may cause cerebral edema due to BBB damage and increased fluid permeability through the barrier [8,9,10,11].

Animal studies have shown that the increased transmissibility of the BBB has been caused by abnormally higher activity and expression of matrix metalloproteinases, such as metalloproteinases 2 (MMP-2) and metalloproteinases 9 (MMP-9). Significant increases in MMP-9 activity were also found in individuals who had a previous stroke [12,13,14]. Panickar’s studies [15] have shown that MMP-9 modified the permeability of BBB by the degradation of its components, such as tight junction proteins including occludin and claudin-5. Occludin is critical to the barrier function at the site of the tight junctions between microvessel endothelial cells. Moving outside of the tight junctions is a sensitive, early, and reliable signal of the opening of the tight junctions and BBB dysfunction [16]. Aquaporin-4 plays an important role in the pathogenesis of cerebral edema, as its molecule is the most efficient aqueous channel in this area, regulating its transmembrane flow in astrocytes [17].

S100β protein is one of the best-known biomarkers of central nervous system damage. The low molecular weight of S100β protein causes it to permeate the blood–brain barrier even with a minor pathology, and even a relatively small activation of astrocytes increases its synthesis and penetration into the periastrocytic space. Elevated blood concentrations observed in stroke patients may indicate an ongoing release of the marker by necrotic tissue [18].

The objective of this study was to evaluate the activity of MMP-2 and MMP-9 as well as the concentrations of claudin-1, occludin, aquaporin-4, and S100β protein in the blood of patients undergoing extracranial carotid reconstructive surgery.

## 2. Materials and Methods

The study was conducted based on the approval of the Bioethics Committee (KE-0254/146/2010).

A total of 88 elective patients were enrolled in the study due to extracranial stenosis of the internal carotid arteries, which qualified for interventional treatment according to the guidelines of the European Society for Vascular Surgery [19] based on Doppler testing performed with a Toshiba Aplio 500 instrument. The morphological nature (stability) of the atherosclerotic plaque and the degree of stenosis were evaluated based on the North American Symptomatic Carotid Endarterectomy Trial (NASCET) study recommendations [20]. The mean age of the patients was 67 (53–83) years old. The mean stenosis of the right internal carotid artery was 75% (50–90%), and the left, 80% (50–95%). Patients were divided into two groups depending on the surgical technique used: internal carotid endarterectomy (CEA), 66 patients, and endovascular percutaneous internal carotid balloon angioplasty and stenting (CAS), 22 patients. The mean time of surgery was 67 ± 14 min in the CEA group and 61 ± 10 min in the CAS group, respectively. The cross-clamping time was 11 (5–39) min. No local or systemic surgical complications were observed.

Blood for laboratory assays was drawn from the basilic vein 24 h prior to surgery (measurement 1), 8 h post-surgery (measurement 2), 24 h post-surgery (measurement 3), and 48 h post-surgery (measurement 4). After centrifugation at 3500 rpm for 5 min, the samples were frozen and stored at −80 °C. Samples for assays were thawed in a water bath at 37 °C.

**Figure 1:**
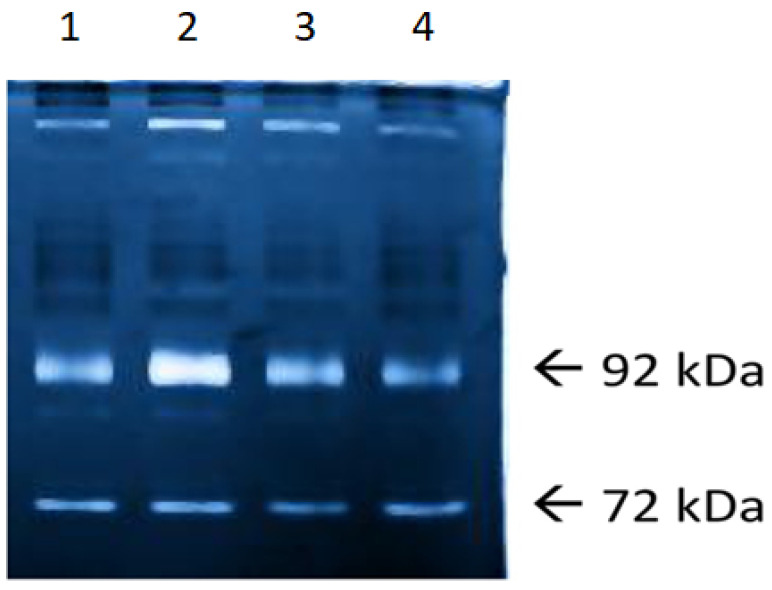
MMP-2, 72 kDa and MMP-9, 92 kDa in the next stages of research (1, 24 h before surgery; 2, 8 h post-surgery; 3, 24 h post-surgery; and 4, 48 h post-surgery).

Claudin-1, occludin, aquaporin-4, and S100B concentrations were assayed with pre-filled reagent sets using the ELISA assay, and MMP-2 and MMP-9 activity was evaluated using zymography. Determination of gelatinases was made using the G BOX gel analysis and documentation system by comparing the locations of the bands obtained both with a protein pattern of known molecular weight and with a location relative to the control MMP-2 and MMP-9. Quantitative analysis was performed using Gene Tools computer software (Figure 1).

The results of the studies were analyzed statistically. Due to the skewed distributions of the tested measurable parameters as assessed by the Shapiro–Wilk test, and the heterogeneity of variance assessed by Levene’s test, to analyze the differences between the studied subgroups nonparametric tests were used. The two independent groups were compared with the Mann–Whitney U test, while more than two independent groups were compared with the Kruskal–Wallis H test and for the dependent groups, Friedman’s ANOVA test and post hoc multiple comparisons were used. The significance level was set at *p* < 0.05. Statistical analyses were performed based on computer software STATISTICA v. 10.0 (StatSoft Inc., Tulsa, OK, USA).

Patient parameter characteristics are presented in Table 1.

## 3. Results

**Figure 2:**
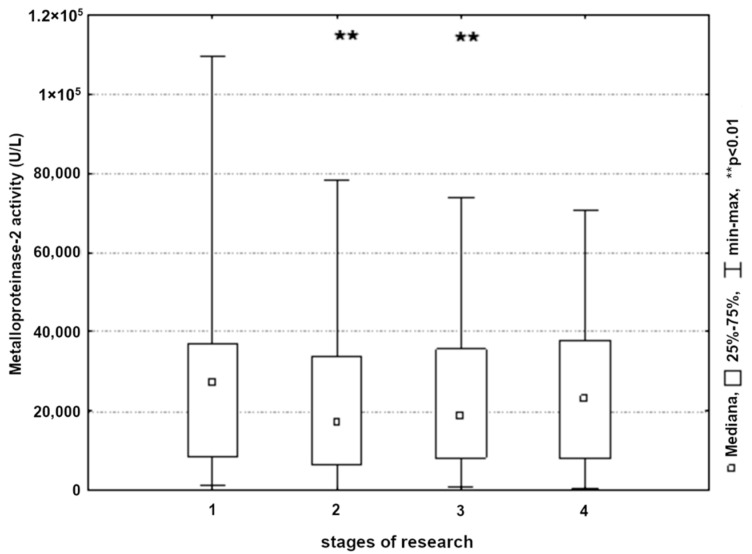
Metalloproteinase-2 activity in patients in the next stages of research (1, 24 h before surgery; 2, 8 h post-surgery; 3, 24 h post-surgery; and 4, 48 h post-surgery).

A significant decrease in MMP-2 activity in the blood was found in all subjects undergoing endarterectomy. Most significant changes were recorded immediately after the surgery and in the morning on the first postoperative day (*p* < 0.01) (Figure 2).

**Figure 3:**
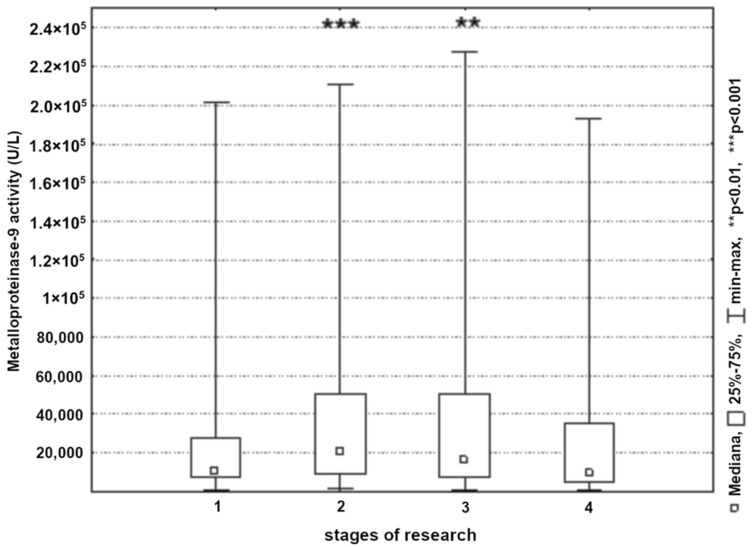
Metalloproteinase-9 activity in patients in the next stages of research (1, 24 h before surgery; 2, 8 h post-surgery; 3, 24 h post-surgery; and 4, 48 h post-surgery).

A significant increase in MMP-9 activity in the blood was found in the endarterectomy group and applied to all studied patients. As with the MMP-2 analysis, the largest changes were observed immediately post-surgery (*p* < 0.001) and on the first postoperative day (*p* < 0.01) (Figure 3).

**Figure 4:**
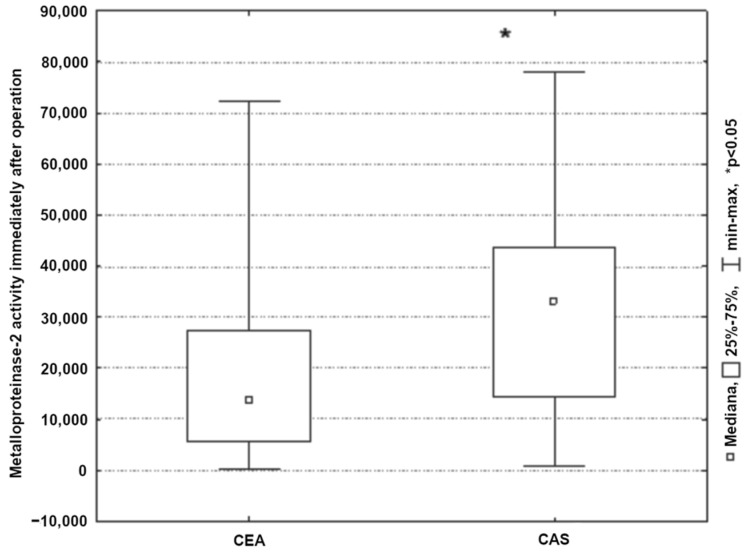
Changes of MMP-2 activity immediately after CEA/CAS procedure.

Analysis of MMP activity 2 and 9 showed no significant differences between patients in the CEA and CAS groups immediately prior to surgery (*p* > 0.05). Significantly higher MMP-2 activity was noted in patients undergoing angioplasty, but only immediately after the surgery (*p* < 0.05) (Figure 4).

**Figure 5:**
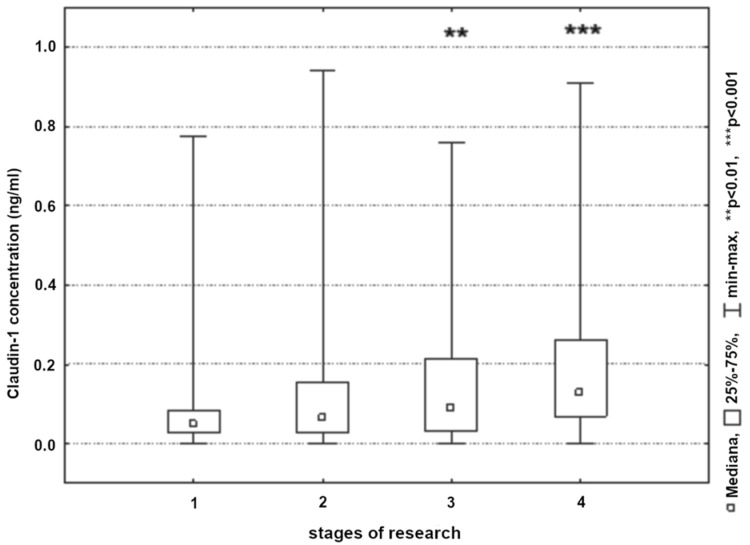
Claudin-1 concentration in patients in the next stages of research (1, 24 h before surgery; 2, 8 h post-surgery; 3, 24 h post-surgery; and 4, 48 h post-surgery).

An analysis of the changes in claudin-1 concentrations in all studied patients showed a significant increase on the first and second postoperative day (Figure 5). Significantly higher claudin-1 concentrations were reported in patients who underwent percutaneous carotid artery stenting (CAS) (*p* < 0.05).

**Figure 6:**
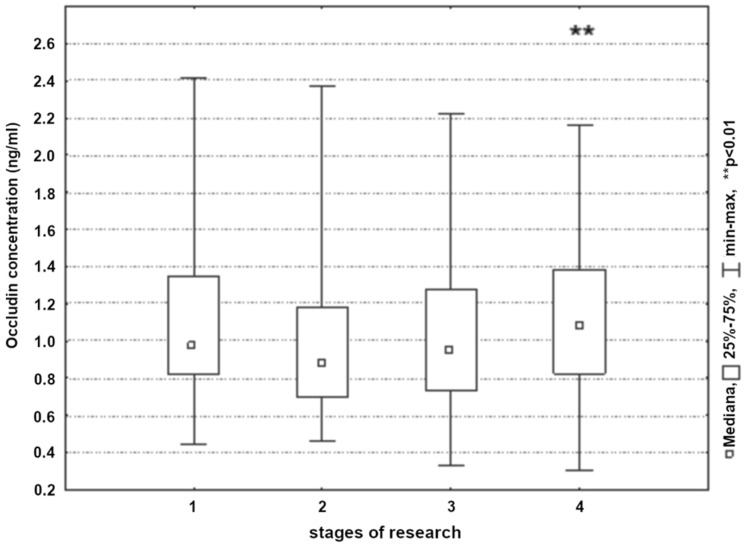
Occludin concentration in patients in the next stages of research (1, 24 h before surgery; 2, 8 h post-surgery; 3, 24 h post-surgery; and 4, 48 h post-surgery).

**Figure 7:**
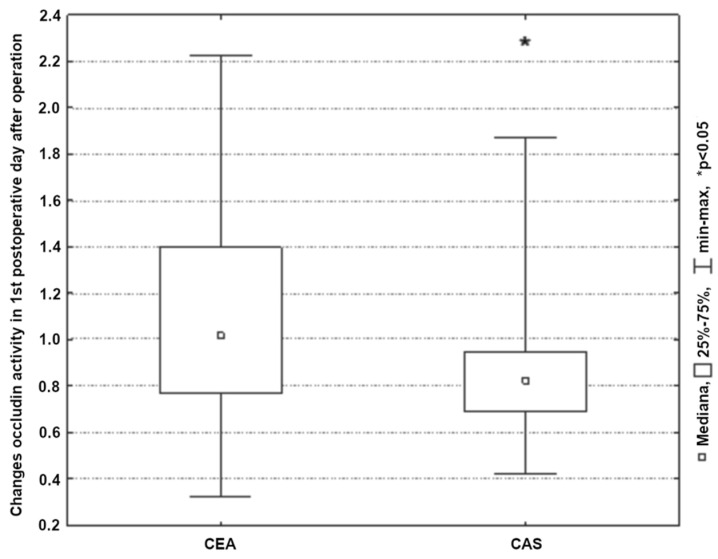
Changes occludin activity in 1st postoperative day after CEA/CAS procedure.

A significant increase in occludin concentrations was observed in all subjects only during the near-postoperative period (*p* < 0.01) (Figure 6). On the first and second postoperative days, regardless of the type of procedure performed, the concentration of the tested parameter was close to the baseline values. Significantly higher occludin concentrations were found on the first postoperative day (*p* < 0.05) in the group of patients who underwent carotid endarterectomy (Figure 7).

**Figure 8:**
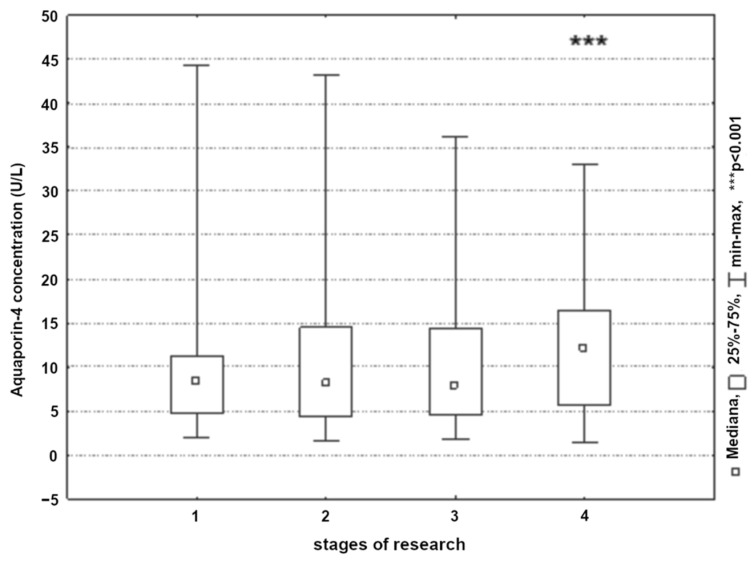
Aquaporin-4 concentration in patients in the next stages of research (1, 24 h before surgery; 2, 8 h post-surgery; 3, 24 h post-surgery; and 4, 48 h post-surgery).

Cerebral circulatory revascularization resulted in a significant increase in blood plasma aquaporin-4 concentration in both groups. Most significant increases/changes were recorded on the second postoperative day (*p* < 0.001) (Figure 8). An analysis of the aquaporin-4 concentration based on the type of surgery did not show statistically significant differences between the studied groups (*p* > 0.05).

**Figure 9:**
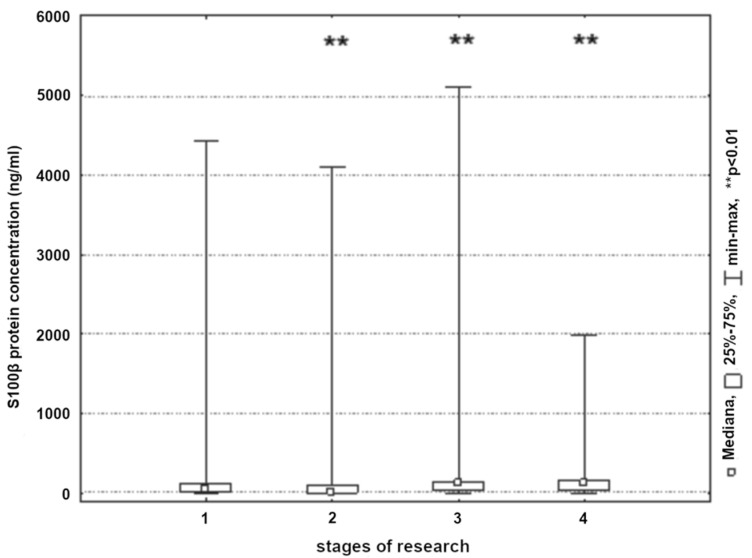
S100β protein concentration in patients in the next stages of research (1, 24 h before surgery; 2, 8 h post-surgery; 3, 24 h post-surgery; and 4, 48 h post-surgery).

Both studied groups showed a significant reduction in the concentration of S100β protein within a short time after the procedure (*p* < 0.01). Within the first postoperative day, the concentration of S100β protein increased significantly and then decreased on the second postoperative day (*p* < 0.01) (Figure 9, Table 2). Significantly higher S100β protein concentrations were reported in the group of patients undergoing endarterectomy before and immediately post-surgery (*p* < 0.05).

No statistically significant differences were found with respect to patient demographic data (*p* > 0.05) when analyzing the changes in the tested parameters depending on the stability of the atherosclerotic plaque. Significantly higher MMP-9 activity was found prior to surgery in the group of patients with unstable atherosclerotic plaque (*p* = 0.05), and the MMP-2 activity was comparable across all groups and throughout the observation period. In the group of patients with unstable atherosclerotic plaque, significantly higher S100β protein concentrations were found prior to surgery (*p* < 0.05). The concentrations of the remaining tested parameters were similar in the group of patients with stable and unstable atherosclerotic plaque (Table 3).

## 4. Discussion

Open carotid endarterectomy (CEA) is an effective treatment for internal carotid artery stenosis in its extracranial segment. It is the basis for stroke prevention in patients with symptoms of cerebral ischemia as well as in asymptomatic patients. The alternative for this treatment is percutaneous angioplasty and stenting; however, numerous clinical studies conducted to date have indicated that the surgical method has been associated with fewer serious perioperative neurological complications [21,22,23,24]. Wu et al. reported that early revascularization, including revascularization within seven days, may be performed safely. It was considered to be a better course of management than the delayed surgical treatment of symptomatic carotid stenosis in patients without signs of hemorrhagic stroke, carotid artery occlusion, or deep neurological deficit [25]. This has also been confirmed by the authors’ experience in 20 endarterectomies and thrombectomies or embolectomies performed during the “stroke in evolution” period [26]. In Rapp’s study [27], which analyzed 54 internal carotid angioplasty procedures with excellent clinical results, a significant number of new lesions in diffusion weighted magnetic resonance imaging (DW-MRI) were found. These subclinical brain injuries did not occur during the procedure but in the subsequent 48 h when transcranial Doppler examinations confirmed an active embolic process. According to Feiwell, new lesions were rarely observed in MRIs after endarterectomy [28]. Comparing the CAS and CEA procedures, Roh and Szarmach [29,30] demonstrated that both neurological events as well as new lesions in the MRI radiographic image were more common in patients undergoing endovascular procedures.

Ischemia and hypoxia of the central nervous system trigger a number of events leading to the interruption of occluding junctions and increased BBB permeability. They appear to be mediated by cytokines, vascular endothelial growth factor (VEGF), and nitric oxide (NO). Following focal and generalized cerebral ischemia in animals and in cerebrospinal fluid, post-stroke patients demonstrated elevated concentrations of proinflammatory cytokines, IL-1 and tumor necrosis factor (TNF). In the experimental BBB in vitro model comprising human endothelial cells and astrocytes, it was found that induced ischemia stimulated the secretion of IL-8 and MCP-1 from endothelial cells and astrocytes [31,32,33]. Oxidative stress damaged endothelial cells forming the BBB and contributed to vasogenic edema. The superoxide radical (O^2−^) was identified as the primary reactive form of oxygen contributing to increased blood vessel permeability and the formation of edema in generalized and focal cerebral ischemia [34]. BBB damage is a progressive process. Initial ischemia injury progresses through ischemia via a reperfusion mechanism [35]. BBB damage associated with reperfusion has been the subject of extensive research, which has led to the identification of multiple mechanisms that contribute to the reperfusion damage of the BBB, such as oxidative trauma, inflammatory damage, vascular activation, or dysregulation of extracellular proteolysis. Unlike the reperfusion phenomenon, the initial stage of damage to the BBB resulting from ischemia has been a less studied topic [36]. Many studies using the BBB in vitro model have demonstrated that hypoxia and hypoxia/reoxygenation leads to increased permeability and/or a rupture of BBB-occluding junctions [37,38,39], although the stress associated with hypoxia may also increase transcellular permeability [40]. In vivo hypoxia has been associated with increased BBB permeability, reduced occludin expression, and the activation of nuclear transcription factors NF-kB and hypoxia-induced factor [41].

Numerous studies have suggested that MMP-9 played a key role in the modulation of BBB permeability in various conditions such as head injury, acute liver failure, focal or generalized ischemia, and reperfusion in the central nervous system. Yang’s studies have shown that the development of cerebral edema and increased BBB permeability in the early period following an endarterectomy of the internal carotid artery and the restoration of normal circulation resulted in increased MMP-9 activity and expression, and a reduction in the number of proteins in tight junctions, including claudin-5 and occludin. It was established that the disturbance of the structure of the tight junctions in the barriers was directly related to the pathogenesis and worsening of cerebral ischemia and post-traumatic reperfusion [42]. MMP-9 has been linked to BBB permeability by modulating the proteins of the tight junctions and basal plaque combinations, which confirmed that the increased MMP-9 expression was accompanied by a reduction in basal lamina proteins including laminin and fibronectin. That literature has also suggested that increases in MMP-9 activity and its expression correlated with a decrease in claudin-5 and occludin levels, suggesting that the regulation of BBB permeability by MMP-9 may occur by the degradation of the tight junction proteins. Given that the matrix metalloproteinases are a family of enzymes that are activated by oxidative stress, and ischemic post-conditioning may inhibit oxidative stress caused by the release of the carotid stenosis, we speculated that the inhibitory effect of ischemic post-conditioning on MMP-9 could be caused by blocking oxidative stress, which was also confirmed by studies where the administration of a free radical scavenger inhibited BBB damage [43,44]. MMP-2 is the primary gelatinase secreted by ischemic cerebral tissue two hours after middle cerebral artery occlusion (MCAO), which was consistent with the assumption that MMP-2 was an early mediator in ischemic brain injury, and MMP-9 was an inflammatory molecule stimulated and involved in brain damage at relatively late stages of stroke [45]. The MCAO in vivo model also showed that cerebral ischemia rapidly induced the degradation of occludin and redistribution of claudin-5 in ischemic cerebral microvasculature [46].

The authors’ research has shown a significant increase in MMP2 and MMP9 activity and a significant increase in the concentrations of claudin-1 and occludin in the blood of patients undergoing the reconstruction of internal carotid artery stenosis. Due to the involvement of the above parameters in the damage to the blood–brain barrier and cerebral ischemia, the results obtained suggested the occurrence of the above processes in the studied patients.

S100B protein has been considered a sensitive marker of cerebral trauma [47,48]. In the periodical Doppler (TCD) examination, during carotid revascularization procedures, a significant number of high intensity transient signals (HITS) consistent with embolisms correlated with S100β concentration changes were found. The CAS procedure has been associated with a higher number of perioperative HITS, as compared to the CEA procedure (despite the routine use of neuroprotection systems); however, no statistically significant changes were observed in S-100b concentrations when comparing both methods. A statistically insignificant, but apparent, tendency was observed for the mean concentration of S100β to increase in patients undergoing the CAS procedure, while the mean concentration of this marker in patients undergoing CEA remained constant throughout the duration of the procedure [49]. Capoccia et al. reported a postoperative increase in the concentration of S100β protein and neuron-specific enolase (NSE) in patients post-CAS and in all those demonstrating new lesions in the postoperative DW-MRI examination. These changes correlated with significant decreases in postoperative MMSE neuropsychometric tests with a tendency for some improvement at the six-month follow-up. In addition, a significant relationship was noted between the emergence of new ischemic lesions in the postoperative DW-MRI examination and the deterioration of MMSE results in patients post-CAS, as compared to preoperative values and the results of patients who did not demonstrate postoperative ischemic lesions [50].

Our research showed a statistically significant increase in S100β protein concentrations in the patients’ blood within the first postoperative day. Significantly higher concentrations of this parameter were observed in patients treated with endarterectomy prior to and immediately post-surgery, which may indicate cerebral ischemia following surgery.

Cerebral edema is considered an important factor of high morbidity and mortality due to extensive ischemic strokes. Solenov et al. demonstrated that astrocytes exhibiting aquaporin-4 deficiency were seven times less permeable to water as compared to identical cells with normal aquaporin-4 expression [51]. The exclusion of aquaporin-4 and the interference of the expression of its polarized parts reduced the accumulation of fluid in the brain associated with ischemia, water intoxication, and hyponatremia, suggesting that under pathophysiological conditions, aquaporin-4 was the main route of penetration of plasma water into the brain. Aquaporin-4 deficiency reduced cytotoxic edema caused by water intoxication, cerebral ischemia, and acute bacterial meningitis; however, it intensified vascular edema caused by brain tumor, the damage to the cortex due to hypothermia, central nervous system infections, and kaolin-induced obstructive hydrocephalus [52,53]. The data on the expression of aquaporin-4 in cerebral edema, particularly aquaporin-1 and aquaporin-4 that are expressed in the central nervous system and participate in the transport of water by the BBB, have been taken from several recent studies on extensive ischemic strokes [54]. The presence of aquaporin-4 molecules in the BBB suggested that the aquaporin-4 molecules may be important for maintaining water balance in the brain and may play a key role in the pathogenesis of its edema. Aquaporin-4 is found in the astrocytic processes between the brain tissue and larger fluid compartments, including spaces containing cerebrospinal fluid and blood. The aquaporin-4 degradation indicated that it played an important role in controlling the flow of water into and out of the brain tissue [55]. The importance of aquaporin-4 in the development of cerebral edema was confirmed by studies on the sepsis-associated encephalopathy model, which showed that a higher level of aquaporin-4 was directly associated with a more severe degree of edema [56]. Data from studies of aquaporin-4 conducted in knock-out mice suggested that an aquaporin-4 molecule was involved in the removal of extracellular fluid from brain tissues during vasogenic edema. The aquaporin-4 observations in the knock-out mice with water intoxication and focal cerebral ischemia suggested that during vasogenic edema, water entered the brain tissues regardless of aquaporin-4, but left the brain with aquaporin-4 [57]. The increase in aquaporin-4 concentration observed in the authors’ own research within the second postoperative day suggested significant disturbances in BBB permeability resulting in cerebral edema.

## 5. Conclusions

To summarize, the increase in the concentration of the BBB damage markers and central nervous system damage indicated that carotid endarterectomy may have led to injury in the central nervous system, secondary to damage to the BBB. Both techniques may have led to injury, but the degree of damage to the BBB was dependent on the surgical technique performed, and it was higher in the group of patients treated surgically with CEA.

## Figures and Tables

**Table 1:** Characteristics of parameters in patients.

		Numer of Patients	%
Sex	Female	21	23.9
	Male	67	76.1
Right carotid artery stenosis	Absent	44	50.0
	Present	44	50.0
Left carotid artery stenosis	Absent	19	21.6
	Present	69	78.4
Symptoms	Stroke in anamnesis	29	33.0
	TIA in anamnesis	20	22.7
	No symptoms	39	44.3
Operated side	Right	33	37.5
	Left	55	62.5
Type of surgery	CEA	66	75.0
	CAS	22	25.0
Plaque stability post-surgery	Yes	70	79.5
	No	18	20.5

**Table 2:** Comparative analysis of the concentration of the S100β protein according to the type of surgery.

Stages of Research	Sum.Rang CEA	Sum.Rang CAS	U	Z	n CEA	n CAS	Statistical Significance
1	2694.00	1222.00	483	−2.33	66	22	*p* = 0.02
2	2661.50	1254.50	450	−2.65	66	22	*p* = 0.01
3	2791.50	1124.50	580	−1.40	66	22	*p* = 0.16
4	2781.00	1135.00	570	−1.50	66	22	*p* = 0.13

**Table 3:** Analysis of claudin-1, occludin, aquaporin-4, S100β protein, MMP-4, and MMP-9 concentrations according to the stability of the atherosclerotic plaque (S—stable plaque, U—unstable plaque).

	Stages of Research	Plaque	Median	Interquartile Range	Statistical Significance
Claudin-1 (ng/mL)	1	S	0.05	0.03–0.08	*p* = 0.73
		U	0.06	0.02–0.11	
	2	S	0.06	0.03–0.16	*p* = 0.56
		U	0.06	0.02–0.14	
	3	S	0.09	0.03–0.20	*p* = 0.97
		U	0.09	0.03–0.26	
	4	S	0.13	0.07–0.29	*p* = 0.23
		U	0.11	0.06–0.17	
Occludin (ng/mL)	1	S	0.95	0.81–1.36	*p* = 0.41
		U	1.05	0.94–1.29	
	2	S	0.88	0.7–1.18	*p* = 0.61
		U	0.92	0.75–1.35	
	3	S	0.94	0.7–1.34	*p* = 0.49
		U	1.05	0.83–1.26	
	4	S	1.05	0.81–1.36	*p* = 0.31
		U	1.1	0.94–1.45	
Aquaporin-4 (U/L)	1	S	7.98	5.11–10.59	*p* = 0.85
		U	8.67	3.92–11.85	
	2	S	7.46	4.31–14.29	*p* = 0.38
		U	12.85	4.24–16.9	
	3	S	7.11	4.37–13.96	*p* = 0.19
		U	10.69	5.28–15.55	
	4	S	10.98	5.5–15.76	*p* = 0.2
		U	15.86	6.37–20.6	
S100β protein (ng/mL)	1	S	36.24	13.35–119.7	*p* = 0.02 *
		U	4.98	2.06–105.8	
	2	S	26.82	6.4–108.49	*p* = 0.07
		U	6.49	3.43–32.3	
	3	S	109.39	59.28–146.54	*p* = 0.95
		U	112.08	26.96–153.74	
	4	S	117.94	33.1–169.48	*p* = 0.26
		U	63.37	4.67–148.6	
MMP-2 (U/L)	1	S	27,385.83	1475.42–109,346.2	*p* = 0.89
		U	26,377.89	1495.89–72,648.7	
	2	S	18,398.3	368.23–78,037.9	*p* = 0.72
		U	14,402.55	428.91–72,379.6	
	3	S	20,191.14	919.58–73,871.1	*p* = 0.65
		U	14,444.78	1081.33–73,571.4	
	4	S	23,236.04	543.34–70,777.9	*p* = 0.68
		U	21,306.02	1818.70–65,705.8	
MMP-9 (U/L)	1	S	8577.03	1702.92–163,367.7	*p* = 0.05
		U	18,161.18	561.46–201,649.1	
	2	S	18,856.58	1083.04–210,380.5	*p* = 0.13
		U	43,023.42	4508.04–135,847.3	
	3	S	15,813.51	314.43–200,731.2	*p* = 0.36
		U	18,692.94	1367.15–227,834.6	
	4	S	7932.61	396.33–138,148.9	*p* = 0.1
		U	24,738.39	2729.86–193,071.7	

* Statistical significance.

## Data Availability

Not applicable.
